# Author Correction: The anatomy of a population-scale social network

**DOI:** 10.1038/s41598-023-38796-1

**Published:** 2023-07-18

**Authors:** Eszter Bokányi, Eelke M. Heemskerk, Frank W. Takes

**Affiliations:** 1 University of Amsterdam, Amsterdam, The Netherlands; 2 Statistics Netherlands (CBS), The Hague, The Netherlands; 3 Leiden University, Leiden, The Netherlands

Correction to: *Scientific Reports* 10.1038/s41598-023-36324-9, published online 06 June 2023

The original version of this Article omitted an affiliation for Eszter Bokányi and Frank W. Takes. The correct affiliations are listed below.

Eszter Bokányi

University of Amsterdam, Amsterdam, The Netherlands.

Statistics Netherlands (CBS), The Hague, The Netherlands

Frank W. Takes

Statistics Netherlands (CBS), The Hague, The Netherlands

Leiden University, Leiden, The Netherlands

In addition, Figure 6 in the original version of this Article was published with tracked changes, which have now been removed. The original Figure 6 and accompanying legend appear below.

**Figure 6:**
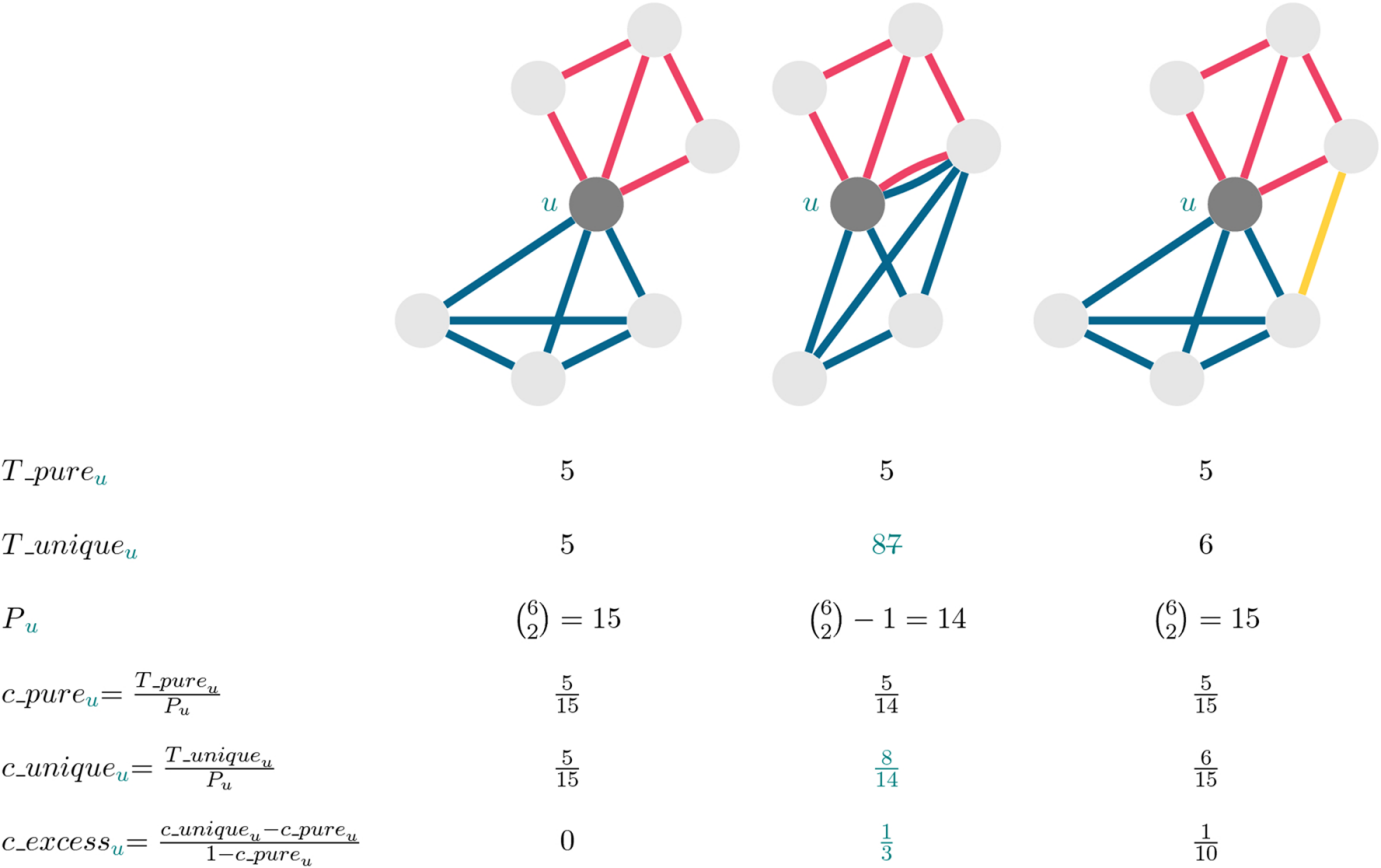
Three sample ego networks illustrating the concept and the calculation of excess closure. Ego node *u* is the central node shaded darker, edges come from three layers color-coded by red, blue, and yellow. The values of $T\_{pure}$ $_u$, $T\_{unique}$ $_u$, $c\_{actual}$ $_u$, $c\_{pure}$ $_u$, *P* $_u$, and $c\_{excess}$ $_u$ are in the table below the figures.

The original Article has been corrected.

